# Characterization, Preconditioning, Safety, and Other Issues of MSC-Derived EVs and Secretome

**DOI:** 10.3390/ijms27041688

**Published:** 2026-02-09

**Authors:** Elena V. Alpeeva, Anfisa S. Ryabchenko, Ekaterina A. Vorotelyak

**Affiliations:** Laboratory of Cell Biology, N.K. Koltzov Institute of Developmental Biology of Russian Academy of Sciences, Vavilov Street 26, 119334 Moscow, Russiavorotelyak@yandex.ru (E.A.V.)

**Keywords:** mesenchymal stromal/stem cells, MSCs, preconditioning, immortalized MSCs, exosomes, extracellular vesicles, EVs, secretome

## Abstract

Recently, a growing number of scientific research and clinical studies have demonstrated the potential of extracellular vesicles (EVs) secreted by cells of different types for treating various diseases. It was shown that most frequently, substances and molecules excreted by the cells exert therapeutic or other effects, and not the cells themselves. Their cargo is wrapped in membrane envelopes, allowing it to survive for a longer time and find targets in organs and tissues as well as overcome various barriers, including the blood–brain barrier. EVs from mesenchymal stromal (stem) cells (MSCs) have attracted particular interest, as MSCs possess immunomodulatory and tissue-repairing properties per se. However, their clinical use is severely limited due to the frequent lack of efficiency in clinical trials, as well as existing risks of tumorigenesis and pulmonary embolism. EVs isolated from MSCs may help circumvent these problems, but their composition and properties, like those of their progenitors, vary significantly between batches, owing to donor characteristics and cell culture conditions. EVs from immortalized MSCs offer greater potential for repeatability and uniformity but raise the question of whether cell immortalization products enter EVs and are transferred to target cells and/or affect them. This review examines the most recent data on preconditioning techniques for MSC-derived EVs, EV characterization, large-scale manufacturing, storage, and the use of EVs from immortalized MSCs, including their characteristics and therapeutic properties, with a special emphasis on safety issues.

## 1. Introduction

In adult organisms, stem cells play essential roles in sustaining tissue homeostasis and contributing to the repair of the tissues in which they reside [1]. Mesenchymal stem cells (MSCs) are undifferentiated, multipotent, non-hematopoietic cells capable of self-renewal and multilineage differentiation [2], and they are found across a variety of tissues and organs. MSCs can be isolated from multiple mesoderm-derived tissues, including adipose tissue, bone marrow, dental pulp, peripheral blood, Wharton’s jelly, amniotic fluid, placenta, and others [3].

According to the criteria set by the International Society for Cell and Gene Therapy (ISCT)^®^, MSCs are defined by the expression of CD73, CD90, and CD105, the absence of hematopoietic lineage markers, such as CD45, CD34, CD14 or CD11b, CD79α or CD19, and HLA-DR, and the capacity to adhere to plastic surfaces during in vitro expansion [4].

MSC biology has been widely investigated due to its immunomodulatory capabilities, specifically, its ability to inhibit the proliferation of T-, B-cells, and Natural Killer (NK) cells [5,6], its anti-inflammatory activity, and its capacity to stimulate endogenous tissue repair through differentiation and paracrine signaling. Moreover, low levels of the major histocompatibility complex (MHC) class I and HLA-I and no expression of HLA II and co-stimulatory molecules (e.g., CD40, CD80, and CD86) reduce their immunogenicity, providing advantages for allogeneic transplantation [7,8]. As a result, MSCs hold substantial promise for clinical application [9].

A pharmiweb.com report “Mesenchymal Stem Cells/Medicinal Signaling Cells–Advances & Applications, 2025” (published October 9, 2025) notes “1670+ Trials, 12 Approvals Worldwide” (https://www.pharmiweb.com/press-release/2025-10-09/mesenchymal-stem-cellsmedicinal-signaling-cells-advances-applications-report-2025-1-670plus-trials (accessed on 1 February 2026)). Thus, we notice that despite the large number of completed clinical trials and the well-established safety profile of MSCs, only 12 MSC-containing products have received global regulatory approval to date [10]. Limited therapeutic efficacy observed in clinical trials and the pronounced heterogeneity of primary MSC (pMSC) products contribute to this slow translation. Such heterogeneity arises from donor-related factors (health status, genetic background, sex, age) [11]; differences in stemness stability and differentiation potential across MSC sources (e.g., bone marrow, adipose tissue, umbilical cord, etc.); and variation in expansion capacity under diverse culture conditions (confluence, substrate, oxygen tension, vessel type, passage number, surface modifications) [12]. Additional challenges at the application stage include variability in MSC homing and migratory behavior depending on administration route, injection site, infusion conditions, and carrier materials; donor–recipient immune compatibility influenced by inflammatory cues capable of inducing MHC-II expression; and the dynamic composition of MSC-secreted bioactive factors shaped by the host microenvironment (inflammation, hypoxia, extracellular matrix (ECM)), which generate functional variability [12].

Although MSCs were initially viewed as stem cells exerting therapeutic effects through engraftment and differentiation, current evidence indicates that their primary mode of action is paracrine signaling. This shift has led to the proposal that MSCs may be more accurately described as “mesenchymal stromal cells” or “medicinal signaling cells” [13]. Rather than directly replacing damaged cells, MSCs are now believed to predominantly act through secreted bioactive factors that attenuate injury and support tissue repair [13,14]. Furthermore, their limited engraftment and short persistence after transplantation constrain cell replacement-based therapeutic strategies [15].

Subsequent studies have shown that the paracrine activity of MSCs is largely mediated by extracellular vesicles (EVs). Camussi and colleagues first proposed that MSC paracrine agents were microvesicles (80–1000 nm) [16], whereas Lai et al. identified these particles as exosomes (~100–130 nm) [17]. Both are now classified as EVs, which have been widely studied for their physiological and pathological roles [18,19]. Current consensus recognizes that small EVs (sEVs; 50–200 nm) are the principal mediators of MSC therapeutic effects [20]. According to the 2024 Minimal Information for Studies of Extracellular Vesicles (MISEV) guidelines, the terms “microvesicles” and “exosomes” should be avoided, favoring the operational definitions “small EVs” (<200 nm) and “large EVs” (>200 nm) to describe EV subtypes [21]. Notably, MSC-derived EVs (MSC-EVs) exhibit therapeutic efficacy comparable to their parent MSCs [16,22,23,24].

EVs are secreted by nearly all cell types. Compared to MSCs, MSC-EVs demonstrate improved stability, biosafety, and storage properties [25]. Originating from endosomal compartments, small extracellular vesicles (sEVs) possess distinct molecular signatures that enable targeted cellular uptake and transport of bioactive molecules, including proteins, lipids, and nucleic acids, such as miRNAs and mRNAs, which are crucial in mediating therapeutic functions previously attributed to whole MSCs [26,27]. Their selective cargo loading and inherent stability support their use as precision drug delivery platforms with superior delivery efficiency and reduced immunogenicity relative to larger EVs. Numerous investigations have demonstrated that MSC-derived sEVs protect loaded molecules and promote cellular uptake via endocytosis [28]. They can also cross endothelial barriers, such as the blood–brain and blood–retinal barriers, making them suitable carriers for CNS-targeted therapies [29]. Their specificity, customizable nature, and favorable safety profiles render sEVs highly promising tools for personalized medicine [30].

As more and more attention is being attracted to EVs, and they are considered a better alternative to MSCs in clinical practice, we have devoted this paper to the up-to-date studies in the field of obtaining, characterization, scaling up, and safety aspects of MSC-EVs, with the future perspectives and improvements of the techniques connected with their utilization and use.

## 2. The Use of EVs in Clinical Practice

As has been widely established, MSC-EVs exhibit several notable advantages over traditional stem cell-based therapies, positioning them as highly attractive candidates for next-generation regenerative treatments [31,32]. First, their safety profile is substantially improved, as EVs lack the ability to proliferate after administration, thereby markedly reducing concerns related to tumor formation. Their nanoscale size and biological compatibility confer minimal immunogenicity and allow EVs to cross biological barriers, including the blood–brain barrier. They do not induce embolic events or transmit infectious agents. Second, EVs demonstrate excellent storage stability, retaining functional bioactivity during long-term preservation at −80 °C and sometimes remaining resilient to repeated freeze–thaw cycles. Third, EVs support a wide range of administration routes, such as topical, intravenous, oral, and nasal delivery, and can encapsulate therapeutic molecules, enhancing their suitability as targeted drug carriers. Finally, EVs offer greater economic feasibility, as immortalized cell lines can continuously secrete EVs, enabling scalable production, reducing costs, and avoiding the labor-intensive expansion processes required for cloned stem cell populations. Collectively, these features make MSC-EVs particularly compelling for clinical translation. By now, about 50 clinical trials investigating MSC-EVs across diverse disease indications are registered on ClinicalTrials.gov (search term: “mesenchymal stem cell/MSC extracellular vesicles/exosomes”). The review of Zhang et al. provides a detailed overview of the molecular pathways through which MSC-EVs exert therapeutic actions across various disease models, giving the examples of ongoing clinical trials [33]. The authors have summarized current clinical progress and highlighted critical challenges that must be addressed, including standardization of EV manufacturing, enhancement of targeted delivery, and optimization of dosing strategies. Their review emphasizes the exceptional potential of MSC-EVs as inherently bioactive delivery systems compared with conventional stem cells.

## 3. Preconditioning Strategies for MSC-Derived EVs

**Figure 1:**
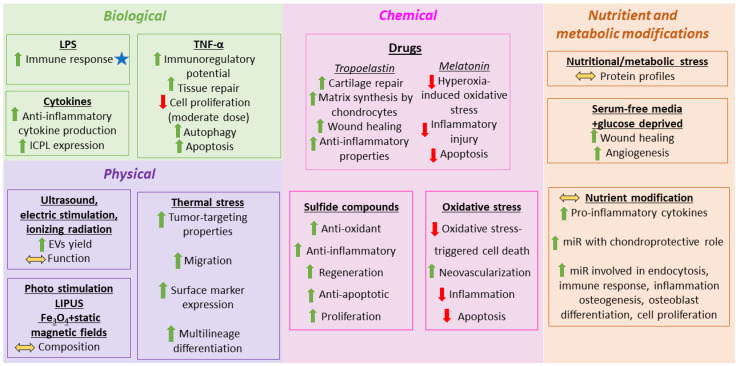
Examples of MSC preconditioning strategies for improving EV properties. The blue asterisk indicates different mechanisms of action depending on the concentration of the modulator; yellow double-headed arrows indicate properties that are dose-dependent; green arrows indicate increasing performance; red arrows indicate decreasing performance.

An area of research that is rapidly evolving focuses on enhancing the therapeutic capacity of EVs by modulating miRNA content through various MSC preconditioning strategies. This approach aims to modify the cellular milieu or apply defined physical stimuli to induce targeted alterations in EV-associated miRNA profiles, thereby improving their regenerative, cytoprotective, and immunomodulatory performance across diverse disease contexts. Based on the review of Song et al., we have highlighted representative examples of different preconditioning techniques on EV properties [34]. Different conditions that influence EV production are summarized in Figure 1.

Different biological modulators affect the properties of EVs. Lipopolysaccharides (LPS) are among the most widely used molecules for MSC conditioning because of their strong immunostimulatory activity. Distinct LPS concentrations induce MSCs to release EVs with different mechanisms of action, which are likely driven by dose-dependent alterations in EV miRNA cargo [35,36,37]. Inflammatory cytokines and growth factors may strongly influence EV production. MSCs contribute to immune regulation by producing immune checkpoint proteins (ICPs) and immune checkpoint ligands (ICPLs) and modulating immune cell behavior through both secreted factors and cell–cell contacts [38]. Exposure to inflammatory conditions considerably enhances the therapeutic potency of MSCs, increasing anti-inflammatory cytokine release and upregulating ICPL expression. The dose-dependency and potential synergistic effects of these stimuli merit further study for their therapeutic use. TNF-α profoundly influences MSC fate in the inflammatory microenvironment, triggering functional reprogramming that enhances its immunoregulatory and reparative potential in a dose-dependent manner [39]. However, moderate TNF-α levels may suppress proliferation and activate autophagy or apoptosis, raising concerns about the feasibility of high-dose TNF-α preconditioning [39].

Pharmacological agents were studied as EV property modulators. Tropoelastin (TE) accelerates wound healing and exhibits anti-inflammatory activity [40]. EVs derived from TE-stimulated MSCs provide superior therapeutic efficacy compared to TE alone by maintaining the chondrocyte phenotype in vitro and having the ability of MSCs to secrete EVs, enhancing chondrocyte matrix synthesis and thus promoting cartilage repair in vivo, probably via increased miR-451-5p expression [41]. Melatonin preconditioned MSC-EVs elevate miR-18a-5p levels, suppressing PUM2 and subsequently activating the DUB3/Nrf2/HO-1 signal axis, thereby mitigating hyperoxia-induced oxidative stress, inflammatory injury, and apoptosis in lung epithelial cells [42]. Atorvastatin-preconditioned MSC-sEVs promoted the proliferation, migration, tube formation, and vascular endothelial growth factor (VEGF) level increase in endothelial cells in vitro. In vivo, they were shown to induce angiogenesis and accelerate diabetic wound repair in rats without influencing liver and kidney function. This was achieved primarily through AKT/eNOS pathway activation and upregulation of miR-221-3p [43].

Oxidative and sulfide compounds were also shown to add therapeutic effects to EVs produced by MSCs. sEV miR-21 from H_2_O_2_-stimulated MSCs could be transported to C-kit^+^ cardiac stem cells, where it suppressed PTEN and thereby activated PI3K/AKT signaling, ultimately protecting against oxidative stress-induced cell death. This demonstrates the potential of sEVs as vehicles to enhance C-kit^+^ cardiac stem cell therapy in ischemic myocardium. Similarly, sEVs from adipose-derived stromal/stem cells (AT-MSCs) generated under low H_2_O_2_ conditions improved skin flap survival, promoted neovascularization, and reduced inflammation in ischemia–reperfusion (I/R) injury, demonstrating the utility of customized sEVs for cell-free therapeutic applications in skin flap transplantation [44]. H_2_S-modified MSC-EVs enriched in miR-7b-5p further induced miR-7b-5p expression, which promoted CD45^low^ microglia and CD45^high^ brain mononuclear phagocytes toward a beneficial phenotype, alleviating hypoxia–ischemia-induced cognitive dysfunction in neonatal mice [45].

Unlike conventional in vitro culture conditions, MSCs naturally exist in hypoxic niches in vivo. Mimicking these oxygen-restricted environments (1–5% O_2_) enhances EV production and substantially modifies their biological activity. Hypoxic preconditioning augments the antioxidant, anti-inflammatory, and regenerative properties of MSC-EVs, making it one of the widely studied strategies in regenerative medicine. Nevertheless, within this range, variations in oxygen concentration can lead to different effects on regulating miRNA expression under hypoxic conditions [46]. Hypoxic culturing of AT-MSCs led to major transcriptional remodeling; 3131 mRNAs were differentially expressed (3116 upregulated), whereas hypoxia pretreatment caused downregulation of all seven significantly altered miRNAs and upregulation of 11 piRNAs. This highlights distinct regulatory mechanisms for mRNA and miRNA cargo loading into EVs [47]. In a retinal ischemia model, hypoxia-preconditioned EVs nearly restored retinal function to baseline, prevented ganglion cell loss, and markedly attenuated pro-apoptotic and inflammatory gene expression, significantly outperforming normoxic EVs [41,48].

Modifying nutrient availability in the culture medium markedly affects EV biogenesis and cargo, making it an important research direction. Fitzgerald et al. demonstrated profound differences in clonal expansion, proliferation, differentiation, and immunomodulation of MSCs depending on the medium used, with substantial variability observed in human bone marrow stem cell EV protein composition [49]. EVs from serum- and glucose-deprived (SGD-EVs) human umbilical cord mesenchymal stem cells (hUCMSCs) significantly increased human umbilical vein endothelial cell (HUVEC) migration, proliferation, and tube formation in vitro. In vivo, SGD-EVs accelerated wound closure and enhanced angiogenesis in rats more effectively than control EVs, largely via induction of VEGFA production during wound angiogenesis by miR-29a-3p transfer and activation of Wnt/β-catenin signaling [50]. A common approach is adding platelet lysate and growth factors to the culture medium. Supplementation of the media with platelet lysate under 1% hypoxia for 48 h induced overexpression of miRNAs (miR-23a, miR-125b, miR-199a/b, miR-4454, miR-7975) in different stem cells, including human bone marrow mesenchymal stem cells (hBMSCs), heart-derived cells, and hUCMSCs. These miRNAs are involved in endocytosis, immune response, inflammation, osteogenesis, osteoblast differentiation, and cell proliferation [51]. Xeno-free media (XFM) supports safe expansion of MSCs producing therapeutic EVs, avoiding issues linked to serum-containing media, which makes ready-to-use clinical therapies more accessible. EVs from XFM showed strong anti-inflammatory effects on osteoarthritic chondrocytes and were enriched in chondroprotective miRNAs (let-7b-5p, miR-17, miR-145, miR-21-5p, miR-214-3p, miR-30b/c-5p). MiR-145 and miR-214 were found to suppress IL-1α-induced inflammation and to reduce pro-inflammatory cytokine production for chondrocyte protection.

Multiple physical stimuli, including ultrasound, electrical stimulation, and ionizing radiation, can enhance MSC-EVs or mimetic nanoparticles secretion. This enhancement facilitates large-scale production and modifies biological functionality via differential miRNA expression [52]. Photo stimulation [53], low-intensity pulsed ultrasound (LIPUS) [54], and Fe_3_O_4_ nanoparticles combined with static magnetic fields [55] have all been reported to influence MSC-EV composition. Other modalities, such as mechanical loading and electrical pulses, remain understudied and may represent future opportunities for EV yield and functionality.

Thermal stress, as shown by Rühle et al., markedly increases MSC adhesion, migration, expression of surface markers, and multilineage differentiation, albeit at the cost of reduced proliferation. Importantly, heat-preconditioned MSCs displayed enhanced tumor-targeting properties [56]. The broader impact of thermal or other environmental conditions on MSC-EVs remains poorly defined, underscoring the need for further systematic investigation.

Combining advanced genetic approaches (e.g., gene editing tools, such as CRISPR/Cas9) with established preconditioning strategies may further improve MSC-EV potency, particularly in oncology, where immune checkpoint blockade mechanisms are crucial. Overall, the choice of preconditioning mode depends on therapeutic objectives, desired molecular modifications, and MSC tissue origin. Diverse preconditioning protocols consistently increase the expression of functionally relevant miRNAs in MSC-EVs. Nevertheless, Song et al. emphasize the requirement of a balanced research strategy to avoid disproportionate focus on a limited set of well-characterized miRNAs, ensuring more comprehensive exploration across the field [34].

## 4. Large-Scale Manufacturing of MSC-EVs

Apart from modifying the EV cargo and thus its functionality and therapeutic potential, these interventions have also been reported to induce EV production. For more examples of preconditioning approaches, one can also refer to the review of Ng et al., who consider preconditioning strategies as a tool for stimulating cultured cells to release greater numbers of EVs, thereby enabling large-scale production [57].

The large-scale production of MSC-derived sEVs for clinical translation presents multiple challenges, particularly regarding the definition of critical quality attributes (CQAs) essential for determining product identity and biological potency. An expert panel representing Society for Clinical Research and Translation of Extracellular Vesicles Singapore (SOCRATES), International Society for Extracellular Vesicles (ISEV), International Society of Blood Transfusion (ISCT), and International Society of Blood Transfusion (ISBT) recommended that MSC-sEV characterization rely on several quantifiable parameters, including the concentration of 50–200 nm particles, protein content, abundance of MSC-associated and MSC-nonassociated surface markers, and the membrane lipid-to-protein ratio. Additionally, assessment of biochemical activity, such as the enzymatic function of canonical MSC-sEV markers, like CD73, is advised to support the identity of MSC-sEV products [58].

Despite these efforts, such parameters still fall short of describing the full biochemical and functional heterogeneity intrinsic to MSC-sEVs across different manufacturing processes. A meta-analysis demonstrated that, although MSC-EV preparations share common MSC-typical proteins, substantial proteomic variability persists between laboratories [59]. Differences in MSC-sEV composition depend on a range of upstream variables, including the origin of MSCs, culture media formulations and supplements, differences between two-dimensional (2D) versus three-dimensional (3D) growth systems, and EV enrichment methods. The use of biological supplements, such as fetal bovine serum or platelet lysate, can markedly alter the EV profile [60].

### 4.1. Bioreactor-Based Manufacturing

Standard 2D culture systems yield relatively small quantities of EVs, creating a major bottleneck for the development of EV-based therapeutics. Therefore, efforts to significantly enhance EV production and improve its properties are essential for clinical-scale applications.

Another major aspect of scalable EV production is the implementation of GMP (good manufacturing practice)-compliant, high-yield culture systems. According to Ahmed et al., hollow-fiber bioreactors (HFBs) provide several advantages over conventional systems, including the ability to maintain long-term cultures without passaging, reduced batch variability, and a more physiologically relevant microenvironment, which supports cell–cell communication and functionality [61]. These conditions promote spheroid formation and can reduce both handling time and overall production cost. Previous reports indicate that maintaining 30 million MSCs in an HFB requires approximately fivefold less medium compared to CellSTACK-based systems [62].

Currently, the challenge inherent to HFB systems is the limited ability to visualize and directly monitor cell morphology and physiology in situ. However, metabolic readouts, including glucose consumption and lactate dehydrogenase release, serve as robust indicators of cellular stability during extended culture. The 4-week stability of iMSCs cultured in HFBs is compliant with observations in pMSCs, which also maintain functionality and EV secretion for up to 30 days [63]. Variability observed in other MSC studies [62,64,65] likely reflects donor heterogeneity and differences in bioreactor configurations.

### 4.2. Immortalized Stem Cells as the Source of EVs for Large-Scale Manufacturing

Despite significant advancements that were made in 3D culture techniques to overcome existing limitations, cell immortalization became one of the most robust techniques utilized to achieve the consistent production of EVs on a large scale using the desired cell source. Additionally, immortalization and culturing in bioreactors may be used in combination to increase EV yield and uniformity [63]. Since one of the initial objectives of our review was to find and analyze information regarding the safety of using EVs derived from immortalized MSCs, we would like to dwell on this issue. We have searched PubMed using the keywords “immortalized” and “extracellular vesicles” and identified a small number (approximately 200) of articles discussing EVs and immortalized cells. However, less than half of these articles are related to the production of vesicles from immortalized stem cells. We have carefully reviewed them and found out that these studies were conducted by only a few research groups. Summarizing the results of the studies, we have focused on the immortalization method, testing of immortalized cells for retention of the parent cell properties, tumorigenicity, and, of course, the characterization of the obtained EVs with an emphasis on their tumorigenic capacity. This data is presented in Table 1. The description and conclusions the authors of the studies made are given below (in accordance with the stem cell type that was used for immortalization and further EV obtaining).

#### 4.2.1. sEVs from Placental Chorionic Mesenchymal Stromal Cells (CMSCs)

Tong et al. achieved stable expression of the Simian virus 40 large T (SV40 LT) gene using lentiviral vectors and resistance gene screening, enabling the generation of an immortalized CMSC line (imCMSCs) [66]. SV40 LT inactivates p53 and pRb, allowing cells to bypass cell cycle arrest, enter the S phase, and modulate telomerase to preserve telomere length, thus enabling long-term proliferation while maintaining cellular functionality [81].

At passage 50, both imCMSCs and their secreted sEVs retained phenotypic and functional characteristics comparable to parental primary CMSCs and their corresponding sEVs. The immortalized cells preserved the stromal cell surface marker profile and maintained multipotent differentiation capacity. To evaluate genomic stability associated with SV40 LT integration, the authors performed gene mapping and comparative karyotyping of 50th passage imCMSCs relative to CMSCs. Both lines maintained normal chromosomal structures with no detectable abnormalities. imCMSCs exhibited increased telomerase activity without displaying pro-tumorigenic behavior in vitro, as was demonstrated by a soft agar colony formation assay. sEVs isolated from 50th passage imCMSCs were compared with those from third passage parental CMSCs and showed similar morphology, particle number, and protein marker expression. The total EV yield per volume of culture supernatant remained stable across both cell types. The authors additionally assessed the concentration and molecular composition of sEV subpopulations from conditioned media. Minor variations were observed in CD63-, CD81-, and CD9-positive subpopulations when comparing imCMSC-sEVs with those from parental CMSCs, but these differences were not significant. Under equal seeding densities, the total EV output per milliliter was comparable between the two cell sources. Although imCMSCs demonstrated no tumorigenic potential in vitro, the authors noted that the long-term safety of their sEVs in humans and animal models warrants further investigation [82].

#### 4.2.2. sEVs from MYC-Immortalized ESC-Derived Monoclonal MSC Line (E1-MYC)

Chen and colleagues examined the E1-MYC clonal line, an MYC-immortalized MSC subtype [67]. E1-MYC cells demonstrated accelerated proliferation and enhanced telomerase activity while retaining the parental karyotype. sEVs collected before and after immortalization were highly comparable. Biochemical assays, mass spectrometry, Western blotting, and antibody arrays confirmed the presence of membrane lipids, such as cholesterol, sphingomyelin, phosphatidylcholine, and a proteome of approximately 1000 proteins, including canonical sEV markers CD81, CD9, and ALIX [83,84,85]. sEVs also contained diverse RNA species, as determined via array hybridization, RT-PCR, and RNA sequencing [86,87]. Transmission electron microscopy (TEM) and immunogold labeling confirmed the presence of ~100–200-nm vesicles expressing membrane CD81 [14,86].

This scientific group had previously reported that MYC protein was not detectable in either E1-MYC cell secretions or their sEVs, and that sEVs were indistinguishable pre- and post-immortalization, indicating that MSC-sEVs do not transfer MYC oncoprotein capable of enhancing tumor growth [88]. The authors investigated the tumorigenicity of E1-MYC cells and evaluated whether sEVs derived from these cells influenced tumor progression. Anchorage-independent growth was assessed in soft agar, and tumor formation was evaluated in athymic nude mice. Additionally, the in vivo effects of E1-MYC-sEVs were tested using a FaDu head and neck cancer xenograft model [69]. The literature reports controversial findings regarding the influence of MSC-sEVs on cancer progression. Some studies show inhibition of tumor proliferation [4,89,90], whereas others suggest promotion of tumor growth and metastasis [91,92]. In contrast, sEVs derived from MYC-immortalized E1-MYC cells neither inhibited nor enhanced tumor growth in the presented xenograft model.

Because oncoproteins (in contrast to oncogenes) cannot replicate or amplify, the oncogenic risk associated with EVs derived from MYC-modified cells is minimized. Although lentiviral vectors pose theoretical concerns, the therapeutic product will be the EVs rather than the transduced cells themselves, reducing risks related to viral integration. Moreover, the system described in the current study uses a third-generation lentiviral vector, which also decreases the likelihood of generating replication-competent viral particles. For future clinical-grade manufacturing, the authors propose employing lentiviral systems already evaluated in clinical trials [93], which would further mitigate safety concerns.

A subsequent review of the same scientific group described the results of the studies of sEV preparations for topical use in psoriasis obtained from the above-mentioned well-characterized MYC-immortalized monoclonal cell line [68]. The authors established the mechanism of action (MoA) for the alleviation of psoriatic IL-17. They also described quantifiable identity and potency metrics that could be used to define the identity and potency of MSC-sEVs for psoriasis, as outlined in their previous report on their efficacy in alleviating complement-activated neutrophils [94]. The MSC-sEVs did not carry MYC protein or promote tumor growth and were used in the recently completed phase 1 clinical trial (NCT05523011) in a Safety and Tolerability Study of “MSC exosome ointment” (https://clinicaltrials.gov/).

#### 4.2.3. sEVs from Conditionally Immortalized Human BMSCs via Doxycycline-Regulated SV40 LT

In the study of Liao et al., a reversible immortalized BMSC line was established using a doxycycline-inducible (Tet-On) SV40 LT expression system [69]. These cells remained stably immortalized under doxycycline exposure, and immortality could be reversed upon its withdrawal.

The Tet-On system enabled tight control over SV40 LT expression, allowing precise regulation of proliferation by adjusting medium composition. iMSCs retained classical MSC surface markers, lacked hematopoietic markers, and demonstrated robust osteogenic and adipogenic differentiation indistinguishable from pMSCs. sEV secretion was also preserved, with sEVs from iMSCs showing comparable to pMSC-EVs morphology, size distribution, marker expression, and molecular content, thus supposing their therapeutic potential. EVs were collected from iMSCs at population doublings (PD) 20, 40, and 60. Increasing PD did not substantially alter EV secretion levels or core molecular cargo.

In functional assays, sEVs from all PDs (20, 40, 60) significantly reduced LPS/ATP-induced inflammation in SV-HUC-1 cells (epithelial cells isolated from the uroepithelium of an 11-year-old male transformed with the SV40 virus). sEVs from PD20 and PD40 displayed suppressive effects similar to pMSC-sEVs. EVs from PD60 retained overall content stability but exhibited slightly reduced protective effects, consistent with the known decline in MSC differentiation potential at high PDs. Intravesical injection of iMSC-sEVs reduced inflammation and pathological alterations in vivo in a mouse model of interstitial cystitis/bladder pain syndrome.

To mitigate oncogenic risks, SV40 LT expression was tightly controlled by doxycycline. Under doxycycline-free conditions used for sEV production, SV40 LT was transcriptionally silenced, ensuring that iMSC-sEVs lacked LT protein. No adverse effects were detected in functional assays, supporting the preliminary safety profile of sEVs. Nevertheless, the authors recognize that comprehensive safety validation, including proteomic, transcriptomic, and epigenetic analysis, remains essential to demonstrate biosafety similarity with pMSC-sEVs.

#### 4.2.4. EVs from hTERT-Transfected Wharton’s Jelly MSCs

This study introduced a workflow for continuous large-scale MSC-EV production by culturing iMSCs in a HFB and evaluated their functional potential in vitro and in preclinical models with a focus on translation and scalability for therapeutic use [63]. The two established iMSC lines were stable, genetically consistent, and met ISCT criteria for MSCs [4]. Unlike the primary parent cell lines, which declined in the proliferative rate at 40 days of culturing, iMSCs proliferated up to 180–240 days. Both iMSC lines maintained primary MSC marker expression, and their immunomodulatory potential remained unchanged, comparable to pMSCs derived from the corresponding donor, as demonstrated by T-cell suppression assays. These results agree with previous studies of iMSC line properties [95,96,97]. After 30 days of culturing inside a HFB, the cells maintained MSC marker expression. The most expressed EV tetraspanin was CD9, especially in the 2D samples, which was predominant over the other tetraspanins. In HFB-derived samples, the authors observed an increased expression of CD63 alone or in co-expression with CD9. EVs harvested from the HFB showed pro-angiogenic activity, promoting HUVEC tube formation in a dose-dependent manner. High-dose EVs from 3D culture performed almost similarly to those from 2D culture.

#### 4.2.5. Supernatant/EVs from Immortalized AT-MSCs

In the work of Kraskiewicz et al., supernatants from immortalized AT-MSCs (HATMSC) were produced in a serum-free medium under hypoxia, and their content was analyzed by a human angiogenesis antibody array. No significant differences were found in the proliferative activity of supernatants obtained from HATMSCs originating from either patients suffering from venous stasis ulcer or healthy donors [70]. A wide panel of angiogenesis-associated cytokines, such as angiogenin, growth-regulated oncogene (GRO), interleukin-6 and -8 (IL-6, IL-8), VEGF, insulin growth factor 1 (IGF-1), and matrix metalloproteinase (MMP), were found in all tested HATMSC supernatants. Moreover, supernatant treatment significantly enhanced the survival of fibroblasts, endothelial cells, and keratinocytes in a chronic wound model in vitro. Importantly, the authors have shown that in in vitro settings, HATMSC supernatant treatment results in superior fibroblast proliferation than in the case of co-culture with HATMSCs themselves. Thus, supernatants exhibited strong therapeutic effects, suggesting that immortalization may alter secretory function, making it more dependent on culture conditions than donor health. Another interpretation of the authors is that patient-derived MSCs retain intrinsic viability in vitro but exhibit diminished in vivo regenerative capability due to chronic pathology.

The same scientific group also tested microvesicles derived from the supernatants of the HATMSCs for their angiogenic properties compared to EVs from a human endothelial progenitor cell line originating from cord blood (HEPC-CB.1) [71]. In HATMSC1-derived microvesicles, normoxia increased expression of IL-6 (8% vs. 2.5%), IL-8 (61% vs. 51.5%), MCP-1 (66.5% vs. 35%), and TIMP-2 (60% vs. 41.5%). Hypoxia, in contrast, elevated angiostatin (22% vs. 15.5%) and RANTES (11% vs. 6%). Other protein levels remained unchanged between conditions. Microvesicles from both HEPC-CB.1 and HATMSC1 lines were enriched in all analyzed pro-angiogenic miRNAs relative to parental cells. Anti-angiogenic miRNAs were abundant but to a considerably lower extent than pro-angiogenic miRNAs. HEPC-CB.1 microvesicles carried higher levels of pro-angiogenic miRNAs, growth factors (basic fibroblast growth factor (bFGF), VEGF, IL-8), and proteins such as MCP-1 and TIMP compared to HATMSC1 microvesicles; however, both sources exhibited comparable pro-angiogenic activity in vitro.

The authors conclude that further work is needed to determine the role of intact microvesicles and their cargo components in all biological processes during tissue regeneration because tissue regeneration involves processes beyond angiogenesis.

#### 4.2.6. hTERT-Immortalized AT-MSCs Adapted to Suspension Culture (S-hMSCs)

Silva Couto et al. demonstrated for the first time hTERT-immortalized human MSCs adapted to suspension culture (S-hMSCs) for eliminating the need for microcarriers or other matrices to support cell growth [72].

Several immunophenotypic differences emerged between S-hMSCs and their adherent counterparts. The suspension-adapted line expressed CD73 and CD105 at levels comparable to primary AT-MSCs and hTERT-MSCs (>90%) while maintaining <5% expression of CD19, CD34, CD45, and HLA-DR. Notably, suspension adaptation probably led to downregulation of the CD90 surface receptor, an integrin associated with cell–matrix and cell–cell adhesion [98], consistent with their non-adherent phenotype. Additionally, S-hMSCs showed upregulation of CD14, typically absent from hMSCs [4]. This receptor plays a critical role in providing host protection against various infections and is involved in host defense as a co-receptor for Toll-like receptor signaling [99,100]. Although the novel S-hMSCs express CD14, the absence of the expression of hematopoietic markers indicates no hematopoietic contamination [101]. Thus, the presented work demonstrates that suspension-adapted immortalized MSC lines may have different immunophenotype characteristics when compared to adherent ones.

RNA-seq analysis revealed that AT-MSCs and hTERT-MSCs were more similar to each other than to S-hMSCs. Additionally, principal component analysis demonstrated high intra-group similarity among hTERT-MSCs and S-hMSCs, highlighting limitations of donor-dependent MSC sources in manufacturing.

#### 4.2.7. sEVs from Disease Condition Serum-Primed hTERT-Immortalized AT-MSCs

Choi and colleagues obtained sEVs from hTERT-immortalized AT-MSCs purchased from ATCC [73]. Flow cytometry showed the expression of standard MSC surface markers, while the three-lineage differentiation of iMSCs was also preserved. The cells were primed with serum from a non-human–primate model of rheumatoid arthritis (RA) to evaluate the immunomodulatory capacity of exosomes under disease-mimicking conditions. Primed iMSC-sEVs (sEVs-RA) exhibited significantly increased particle yield and higher total CD81 levels compared with sEVs-FBS controls, despite no major differences in the expression of key miRNAs (miR-155-5p, miR-146a-5p, miR-10-5p, miR-142-3p, miR-216a-5p).

Although Treg and Th17 changes were minimal, sEVs-RA administration increased Th2 and M2 macrophage polarization. sEVs-RA also reduced pro-inflammatory cytokines (TNF-α, KC, IL-12p70) and mitigated cartilage damage in the RA model. Overall, disease-condition priming enhanced the functionality of iMSC-sEVs by boosting transforming growth factor beta 1 (TGF-β1) production and shifting immune responses toward anti-inflammatory pathways.

#### 4.2.8. EVs from hTERT-Immortalized AT-MSCs

The immunosuppressive activity of MSCs is largely mediated through their secretome, including IDO-1, PGE2, NO, TGF-β1, hepatocyte growth factor (HGF), and IL-10. IDO-1 is a rate-limiting enzyme in tryptophan catabolism [102] and plays a key role in inducing immune tolerance [103]. Enhancing the therapeutic performance of MSC-EVs remains an active research direction worldwide [104].

Haghighitalab examined the secretory properties of hTERT-immortalized AT-MSCs and further exposed cells to stress stimuli or genetic modifications to identify optimal strategies for obtaining a qualified subtype of immunoregulatory MSCs and generating immunoregulatory sEVs using them [74]. The immortalized cells had the expression pattern of surface markers typical of MSCs. As had been previously shown by Tátrai et al., they had preserved all essential MSC features, had clones with normal karyotype, had not senesced after PD100, and had no signs of in vivo tumorigenicity [75]. Immunocytochemistry revealed robust induction of IDO1 and PTGS2 proteins in response to IFN-γ at 72 h post-treatment, whereas TGF-β1 expression showed minimal change. Western blotting confirmed differential expression of IDO1, PTGS2, and TGF-β1 at various time points following IFN-γ and poly(I:C) exposure. Of note, a remarkable increase in protein levels of IDO1 and PTGS2 was evident for the IFN-γ-treated cells. Gene expression analysis showed that combined transduction of hTERT-MSCs significantly upregulated MYD88, STING, DDX58, IFIH1, and PD-L1, whereas single-gene transductions did not produce significant changes. The authors summarized that sEVs derived from all genetically modified MSCs demonstrated strong immunosuppressive activity, surpassing sEVs from mock-transfected or cytokine-primed cells. Moreover, IDO1 overexpression yielded the most pronounced enhancement in the immunoregulatory activity of parent cells, conditioned media, and sEV preparations.

#### 4.2.9. Secretome of hTERT-Immortalized AT-MSCs

Immortalized cells obtained in another study retained the MSC-specific immunophenotype, demonstrated normal contact inhibition, and readily differentiated towards adipocytes, chondrocytes, and osteocytes [76]. The findings presented by the authors indicate that MSC immortalization does not markedly alter either the qualitative or quantitative profile of the MSC secretome. Introduction of human TERT was shown to delay the emergence of senescence-associated secretory phenotype (SASP) components in the MSC secretome. Importantly, iMSC cultures maintained production levels of key neurotrophic and pro-angiogenic factors, including brain-derived neurotrophic factor (BDNF), VEGF, HGF, uPA, and others, as well as their corresponding mRNA expression across extended passaging, with stability observed up to approximately passages 25–33, depending on the specific iMSC line [77].

Beyond stabilizing the secretome composition, immortalization preserved the pro-regenerative functional properties associated with pMSCs. The authors demonstrated that secretome derived from iMSCs (even at passage 30) was capable of inhibiting fibroblast-to-myofibroblast differentiation in an in vitro fibrosis model, enhancing secretory activity of Leydig cells, and promoting neurite outgrowth in murine dorsal root ganglion (DRG) explants. These effects are consistent with the activity previously reported for early-passage pMSC secretome (5th–9th passages) [105,106,107].

However, the authors note that the maintenance of bioactivity in iMSC secretome beyond passage 30 warrants further investigation. Their earlier studies characterizing these iMSC cultures showed that although TERT overexpression increases proliferative capacity and delays cellular aging, it does not confer true immortality [76]. This limitation becomes evident as cultures display reduced proliferation rates and signs of senescence during later passages.

This study was designed with a special emphasis on the safety issues of the secretome of iMCs. To address potential oncogenic risks, the authors evaluated telomerase content in the iMSC secretome, its impact on the expression of oncogenes and tumor suppressor genes in primary fibroblasts, and its ability to induce their malignant transformation (tested in a soft agar assay). Their data showed that the iMSC secretome lacked detectable telomerase protein or corresponding nucleic acids and did not influence pro- or anti-oncogene expression in fibroblasts, nor did it induce their transformation. Nevertheless, the authors pointed out that further safety assessments should employ more sensitive and specific assays to confirm the absence of the telomerase catalytic subunit or ensure that its concentration remains below permissible levels. In particular, methods capable of measuring enzymatic activity, such as the telomeric repeat amplification protocol (TRAP), may be required.

Despite the comprehensive analyses the authors conducted to investigate safety aspects of the secretome of iMSCs, they noticed that not all aspects of this complex system could be covered. They highlight the need for additional investigations focusing on immunological, toxicological, and broader safety profiles of iMSC secretome, ideally incorporating in vivo models.

#### 4.2.10. sEVs from Immortalized Corneal Stromal Stem Cells (imCSSCs)

Another scientific group transfected primary CSSCs with SV40T [78,79]. Obtained imCSSCs maintained their morphology, MSC surface antigen expression profile, and multilineage differentiation capacity for over 15 passages [79]. The findings of the authors confirmed the presence of EV-specific tetraspanins, markers, and endosomal proteins Alix, TSG101, and Syntenin-1. They indicated that sEVs from imCSSCs could modulate dexamethasone-induced gene expression in human trabecular meshwork (TM) cells, particularly suppressing ANGPTL7 expression, a gene strongly associated with glaucoma. imCSSC-derived sEVs are, therefore, considered promising candidates for ocular therapeutics. Nevertheless, the authors emphasize that further validation using in vivo or anatomically relevant systems is needed to confirm therapeutic potential because their current results stem from an in vitro model only.

#### 4.2.11. Secretome from Hair Follicle-Derived MSCs (HF-MSCs)

According to some authors, hair follicle-derived MSCs present several advantages, as they can be collected non-invasively, repeatedly, and from donors across all age groups [108,109]. Dermal papilla-derived stem cells (DPCs) are located at the follicle base within the dermal papilla, a specialized niche responsible for hair morphogenesis and cycling [110]. HF-MSCs and DPCs, being residents of the dermal papilla, differ in anatomical location and functional attributes. While DPCs also possess regenerative capabilities, their niche-specific functions and gene expression patterns render HF-MSCs more suitable for scalable secretome production and broader regenerative applications [110,111].

In the study of Rossello-Gelabert, comprehensively characterized SV40 LT-immortalized HF-MSCs were generated as a consistent and scalable source of secretome [80]. Secretome profiling confirmed that immortalization preserved and, in some cases, enhanced the functional characteristics of HF-MSCs. Two selected clones displayed shortened doubling times, preserved multipotency, and maintained responsiveness to inflammatory licensing (MSCs exposure to pro-inflammatory cytokines IFN-γ and TNF-α), supporting efficient large-scale production. Notably, both clones secreted key immunomodulatory mediators, inhibited peripheral blood mononuclear cell proliferation, promoted Treg differentiation, and demonstrated strong antioxidant activity that protected skin cells from oxidative and hyperglycemic stress. Collectively, these results indicate that immortalized HF-MSCs retain the principal therapeutic attributes of primary HF-MSCs while providing a standardized platform for secretome-based applications. The authors propose that distinct functional strengths of the clones (one exhibiting enhanced immunoevasive properties and the other showing superior antioxidant effects) may suggest potential for context-specific therapeutic use. The authors conclude that immortalized HF-MSCs constitute a robust alternative to pMSCs, supporting the advancement of safe, scalable, cell-free therapies for inflammatory, degenerative, oxidative stress-related, and regenerative disorders.

## 5. Tracing Techniques for EV Characterization

One of the crucial challenges in EV science is developing tracing methods. Recent advances in tracing techniques for MSC-EVs have significantly improved the ability to characterize their biodistribution and pharmacokinetics in vivo. Li et al. [112] present an in-depth overview of state-of-the-art technologies for tracing MSC-EVs, emphasizing that accurate tracking is essential for understanding the biodistribution, targeting specificity, clearance kinetics, and therapeutic mechanisms of EVs in vivo [112]. These technologies have a critical role in accelerating the safe and effective clinical translation of MSC-derived EV therapeutics. The overview of Li et al. represents a systematic and comprehensive comparison of a broad spectrum of imaging strategies: fluorescence and bioluminescent labeling, radionuclide-based single-photon emission computed tomography (SPECT)/positron emission tomography (PET) tracers, magnetic resonance imaging (MRI) agents, computed tomography (CT), and photoacoustic imaging, highlighting that each modality offers distinct advantages but also critical limitations. For example, fluorescence imaging is easy to implement but suffers from shallow tissue penetration and label dilution over time, whereas PET/SPECT provides high sensitivity and quantitative readouts but requires complex radiolabeling protocols and short tracer half-lives. MRI-based labeling enables deep tissue visualization but often alters EV physicochemical properties, raising concerns about biological relevance. The authors draw attention to the persistent challenge that labeling strategies, such as membrane dyes, nanoparticle tagging, genetic reporters, or covalent chemical modification, can inadvertently modify EV structure, uptake dynamics, or functional cargo.

## 6. Moving Towards a Single-Vesicle Analysis

Another essential issue is the shift from bulk analytical approaches toward high-resolution single-vesicle platforms to enhance the sensitivity and specificity of EV biomarkers. This is addressed in the exhaustive review of Tran et al. [113]. The review covers emerging technologies, such as single-particle tracking microscopy, single-vesicle RNA sequencing, and various nanopore, nanoplasmonic, immune digital droplet-, microfluidic-, and nanomaterial-based techniques. Unlike traditional bulk analysis methods, these methods contribute uniquely to EV characterization. Techniques like droplet-based single EV-counting enzyme-linked immunosorbent assays (ELISAs), proximity-dependent barcoding assays, and surface-enhanced Raman spectroscopy further enhance the ability to precisely identify biomarkers, detect diseases earlier, and significantly improve clinical outcomes. Single-vesicle detection platforms bring several advantages, including the ability to resolve EV heterogeneity, detect rare EV subpopulations, and quantify molecular cargo with higher precision, which are critical for clinical implementation of EV-based assays. The authors discuss the strengths and limitations inherent in each technique and conclude that these innovations provide access to intricate molecular details that expand our understanding of EV composition, with profound diagnostic implications. The review also covers various dimensions of EV heterogeneity, focusing on their biogenesis and secretion, size, shape, content, origin, and functions, which are key components of studies in EV isolation and analytical workflows.

## 7. EV Storage

Ahmadian et al. and Sivanantham et al. have recently summarized data on EV preservation [114,115]. Storage conditions have a significant impact on the integrity and functions of EVs. Storage at −80 °C consistently outperforms higher temperatures in preserving EV concentration, molecular cargo, and morphology, particularly during storage periods exceeding one week. Rapid freezing protocols further support EV stability by minimizing freezing-associated damage, underscoring the importance of prompt transfer to ultra-low temperatures. Conversely, repeated thawing and freezing result in cumulative vesicle damage, reinforcing the need for workflows that limit or completely avoid freeze–thaw cycles. Additionally, maintaining EVs in the physiological biofluid environment rather than in standard buffers enhances stability, emphasizing the protective role of native biochemical conditions. Nevertheless, this refers mainly to EVs obtained from plasma, bronchoalveolar lavage fluid, breast milk, and other biofluids.

Beyond conventional cryostorage, alternative preservation strategies, such as lyophilization and biomaterial-based encapsulation, have emerged as promising approaches to mitigate storage-induced EV degradation. Lyophilization (freeze-drying) combined with appropriate cryoprotectants has been shown to preserve vesicle morphology and cargo for extended periods at room temperature, while hydrogel-based systems provide a protective matrix that enhances EV stability and functionality and enables controlled release. Spray-drying is a single-step method that is easier than freeze-drying. Theoretically, it can be used with various agents and allows adjusting the size of the products, but additional research is required to apply this technique. The importance of optimizing coating properties of the vials for EV storage has also been highlighted, emphasizing the need for studying EV–surface interactions and exploring tube-free storage methods, like lyophilization, to enhance EV storage consistency and analysis. Ahmadian et al. note that limited attention has been given to changes in EV surface charge and aggregation behavior during storage, despite evidence that freezing, thawing, and prolonged storage may alter zeta potential and promote aggregation [114]. For more details on the preservation techniques and the examples of the studies, one can refer to the original reviews [114,115]. The authors of the reviews emphasize that substantial discrepancies remain regarding optimal storage temperatures, durations, and the effectiveness of cryoprotectants, likely reflecting differences in EV isolation methods, source materials, analytical techniques, and experimental design.

## 8. Safety Issues of the Use of EVs and Secretome

### 8.1. The Possibility of Immortalization Product Transfer to EVs and to the Target Cells

There have been conflicting results regarding whether or not immortalization may predispose cultures to a cancerous or malignant phenotype. In contrast to immortalization strategies using SV40 LT or E6/E7, which pose risks of unintended genomic effects, as these genes can interfere with tumor suppressor pathways and induce oncogenic transformation [116,117], hTERT’s focus on telomere length maintenance reduces the risk of unintended alterations to cellular behavior and oncogenic potential [118]. Nevertheless, some studies showed that hTERT immortalization can contribute to genomic instability and promote premalignant phenotypes and cancer invasion [119,120,121]. Whether or not the EVs isolated from immortalized cells exhibit similar effects remains to be explored.

As was stated by Garcia et al., a key manufacturing concern was the potential transfer of hTERT via EVs [63]. EVs in their study were isolated using size-exclusion chromatography (SEC), which provides higher-purity EV preparations with reduced mRNA contamination compared to other approaches [122,123]. By RT-qPCR, the authors detected minimal hTERT expression in EVs comparable to levels seen in pMSC-EVs, diminishing concerns over safety. In contrast, previous studies using ultracentrifugation or precipitation isolations reported substantial hTERT mRNA enrichment in EVs of amniocytes [4], Jurkat cells [124], and tumor-derived EVs [125,126] with demonstrated functional consequences on recipient cells.

This was also confirmed in the work of Liu et al., in which hTERT was overexpressed in HFF-1 cells to generate hTERT-immortalized fibroblasts (hT-HFF cells), and sEVs derived from these cultures were applied to primary human fibroblasts [127]. The authors demonstrated that sEVs from hT-HFF cells contained substantial quantities of hTERT mRNA, which was transferred to recipient cells, contributing to telomere lengthening and attenuation of cellular senescence. Although these results indicate a rejuvenating effect, the long-term consequences remain uncertain. Prolonged or repeated exposure to hTERT-containing sEVs may theoretically promote uncontrolled proliferation or other unintended cellular alterations.

As we see from the previous section, not all researchers paid attention to the safety issues while studying the effects of the EVs/secretomes of the immortalized cells. Nevertheless, several authors, who somehow tested EVs and their parent cells for undesired effects, emphasize that additional studies to confirm the safety of their approaches are needed.

### 8.2. Epigenetic Risks

MSC-EVs carry potential risks that warrant careful examination. One concern relates to oncogenic effects, as EVs transport epigenetic regulators capable of inadvertently supporting tumor growth or metastasis. For example, sEV METTL14 transfer has been implicated in the pathology of acute myelogenous leukemia (AML), where MSC-derived sEVs from AML patients deliver METTL14, altering Rho kinase 1 (ROCK1) expression and promoting leukemic cell proliferation and radioresistance [128]. Furthermore, the oncogenic potential of MSC-EVs is intrinsically tied to the biological state of the parental MSCs. Malignant transformation of MSCs could theoretically result in EV cargo that activates tumorigenic pathways in recipient cells. Nevertheless, current clinical evidence supports a strong safety profile for MSC-based therapies, and no cases of MSC-induced tumorigenesis have been documented to date, suggesting that such risks remain largely hypothetical [129].

In this context, Zhang et al. raise another question of unintended epigenetic reprogramming and transgenerational effects [129]. They consider that the long-term impact of EV-mediated epigenetic modifications on germline integrity and heritable traits remains largely unexplored. Certain DNA methylation and histone modifications induced by EVs may persist beyond the intended therapeutic window, potentially influencing offspring phenotypes or predisposing individuals to age-related diseases.

Nevertheless, Karagyaur et al. emphasize that there is a distinction between immortalized and transformed cell lines [101]. Unlike transformed cells, immortalized cultures retain genetic stability, maintain DNA damage response mechanisms, remain sensitive to contact inhibition, and require growth factors for proliferation [130,131,132]. Though it is well established that transformed (tumorigenic) cell lines can modify their microenvironment toward pro-malignant or pre-metastatic states through the secretion of growth factors and microRNAs [133,134,135,136,137], the iMSC secretome does not necessarily exhibit such unwanted properties, and this issue still needs to be studied in detail.

### 8.3. Challenges in Viral Clearance

Giebel et al. draw attention to the fact that sEVs closely resemble viruses in size and biophysical properties; therefore, conventional viral clearance methods, such as nanofiltration, chromatographic purification, or inactivation via heat or low pH, cannot be applied to final EV products [138]. Nonetheless, viral contamination risks can be effectively mitigated by adhering to the ICH Q5A(R2) guidelines (ICH_Q5A(R2)_Guideline_2023). The main sources of potential contamination include the cell substrates and adventitious viruses introduced during manufacturing. Hence, the authors recommend comprehensive viral testing of the parental cell lines and the incorporation of validated virus removal and inactivation strategies throughout production.

## 9. Concluding Remarks

With ongoing advances, MSC-EVs are poised to transform therapeutic paradigms by enabling safer, multifunctional, and highly specific interventions, ultimately supporting the transition from a disease-centered to a system-regulating precision medicine framework. Nevertheless, the diversity of the EVs and their changing nature, depending on the status and the mode of their parent cells, pose several critical challenges that have to be addressed to implement this promising tool successfully. These are:

- Enhancing the therapeutic potential of the EVs via MSC preconditioning;
- Improvement of consistency and scalability of MSC-EVs for large-scale production via MSCs immortalization and other techniques;
- Development of well-defined storage protocols and advanced techniques for preserving the quality, integrity, and functions of EVs to ensure reproducibility in EV research and reliability in clinical applications;
- Development and improvement of tracing techniques to characterize EV biodistribution and pharmacokinetics in vivo;
- Moving towards single-vesicle analysis to enhance the sensitivity and specificity of EV biomarkers.

In summary, it is imperative to identify a reliable upscaling technique that can produce large quantities of EVs consistently. The perfect choice is when the produced EVs also possess cargo with improved therapeutic potential. Medium composition, supplements, such as serum, platelet lysate, growth factors/cytokines, culture format, oxygen levels, etc., and EV isolation and enrichment methods influence EV yield and quality. Gene editing tools, such as CRISPR/Cas9, may present promising avenues for precisely regulating miRNA content in MSC-EVs along with physical and chemical stimuli. The composition of MSC-EV epigenetic cargo is highly flexible and is shaped by the tissue origin of parental cells, culture microenvironment, and EV biogenesis pathways, resulting in significant heterogeneity that complicates reproducibility and standardization in clinical settings. Increasing evidence demonstrates that EVs carry a wide array of epigenetic modulators capable of exerting context-dependent influences across multiple cell types and organs in vivo. The future advancement of MSC-driven epigenetic therapies requires a detailed understanding of how recipient cells incorporate and maintain signals delivered by MSCs and their EVs. Major challenges include elucidating the specificity, durability, and sensitivity of epigenetic modifications in recipient cells, which necessitates high-resolution molecular analyses to capture dynamic regulatory processes. In addition to cell-autonomous effects, MSC-mediated epigenetic modulation must be considered within broader intercellular communication networks. The actions of MSCs and EVs extend beyond directly targeted cells, spreading through tissues via secondary signaling pathways. Addressing these complexities will require spatial transcriptomic and epigenomic methodologies to define how epigenetically reprogrammed cells coordinate interactions that re-establish tissue homeostasis. As insight into these mechanisms expands, EV-based strategies offer substantial potential to advance regenerative and immunomodulatory therapies through targeted epigenetic regulation.

Robust CQAs for optimization and control of all parameters for a given disease connected to a clearly defined MoA are required to obtain the products that would pass trials and gain market authorization for medical use. This complexity highlights the need for more stringent molecular profiling and consistent functional characterization. At the same time, no single imaging technology currently provides a complete and unbiased representation of EV behavior, and multimodal approaches may be required to overcome these gaps. The field of EV tracing urgently needs standardized labeling protocols validated for safety, stability, and minimal interference with EV biology. Advanced tracking systems are essential for resolving key translational barriers, including optimal dosing, route of administration-dependent biodistribution, organ-specific EV accumulation, and long-term persistence. These insights directly support the development of reliable pharmacokinetic and pharmacodynamic models, which are foundational for clinical progression of MSC-EV therapies. The successful clinical translation of EV-based diagnostics will also require technologies capable of unifying single-vesicle sensitivity with scalable manufacturing, regulatory compliance, and rigorous validation across diverse patient populations. Key technological barriers, such as the need for high-throughput and high-resolution detection tools, robust standardization of EV isolation, preservation and storage methods, and effective integration of EV analytics into clinical workflows, also have to be overcome.

The growing number of studies using EVs from immortalized MSCs of different origins suggests that this is an important step toward bringing this technology to the clinic. Nevertheless, we have reviewed nearly all examples of these studies, and it should be noted that there is still no uniform standard for MSC immortalization. Various methods (Myc-, hTERT-, and SV40 LT-mediated) are used, and researchers are obtaining immortalized cells with varying degrees of susceptibility to senescence and proliferative capabilities, which may depend on the specificity of the cell donor, methodological shortcomings, and other issues. For large-scale EV manufacturing, the properties of immortalized MSCs have to be better defined and standardized. It should also be emphasized that the authors of the studies do not always address the oncogenic transformation of the parent cells themselves or the safety of using their secretome. Some authors note the need to further explore these issues. However, there are some indications that MSC EVs and secretomes are a much safer tool for clinical use than MSCs themselves, despite the fact that reports of unintended effects from MSCs themselves during research and the use for disease treatment are rare. Nevertheless, this area is poorly investigated and clearly requires more definitive answers and evidence.

## Figures and Tables

**Table 1:** Data from the studies on obtaining EVs and secretome from different immortalized stem cells, representing their characteristics, properties, methods of obtaining, and safety investigations.

Parent Cell Name, References	Characteristics of Immortalized Cells, Comparison with Parent Cells	Characteristics of the EVs, Comparison with EVs from Normal Cells	Tumorigenicity Studies of the EV/Author Notes
Human placental chorionic mesenchymal stromal cells (CMSCs) [66]	Transfected with Simian virus 40 large T antigen (SV40 LT); tested on the 50th passage, normal karyotype, MSC properties confirmed, higher telomerase activity; no tumorigenic potential in vitro	Isolated using Polyethylene Glycol 8000 (PEG, #P2139, Sigma-Aldrich, Darmstadt, Germany) precipitation method; immortalized CMSC-sEVs had similar morphology, number, and no significant variations in CD63, CD81, and CD9 markers of vesicle subpopulations	Further investigation is essential
Human ESC-derived MSCs [67,68]	Immortalization via MYC; parental karyotype, telomerase activity, increased MYC protein expression, negative expression of MHC I, smaller size, faster proliferation, reduced plastic adherence, loss of in vitro adipogenic differential potential and contact inhibition; did not engraft and form tumors in immunodeficient mice, did not acquire anchorage-independent growth	HPLC purification; direct comparison was not found; protein cargo includes sEV-associated proteins CD81, CD9, Alix, and Tsg101; EVs from MSCs before and after immortalization reduced infarct size to the same extent in a mouse model of myocardial ischemia–reperfusion injury, therapeutic potency was evident in other preclinical models of diseases, also from other research groups (aging, ischemic heart disease, orthopedic disease, immune disease, and radiation injury), alleviated psoriatic IL-17	Did not inhibit or promote tumor growth (on a mouse model of FaDu head and neck cancer xenograft), MYC protein was not detectable in either the conditioned medium or sEVs; used in a recently completed phase 1 clinical trial (NCT05523011) in a Safety and Tolerability Study of “MSC exosome ointment”
Human BMSCs [69]	Doxycycline-regulated SV40 LT; 60 passages; the overall immunophenotype of iMSCs was similar to that of parental MSCs; iMSCs maintained their differentiation potential up to PD40, and then it gradually diminished; presence of dox exerted a significant inhibitory effect on the differentiation potential of iMSCs	sEVs obtained via ultracentrifugation, sEV isolation performed at 20, 40, 60 PDs; size 100–150 nm, circular, bilayered membrane structures with a diameter of approximately 100 nm under EM; highly comparable expression of TSG101 and CD81, similar expression levels of miRNA 9, miRNA-146a-5p, and miRNA-223-5p, no significant differences in the levels of inflammatory regulators, including IL-10, TGF-β1, and PD-L1	SV40 LT expression was tightly controlled by doxycycline to mitigate oncogenic risks; no adverse effects detected in functional assays
Wharton’s jelly MSCs [63]	hTERT-immortalized 2 lines from different donors; steady proliferative rate (up to 180–240 days (P30), 2–3 doublings/week); partial chromosome 16q duplication (add(16)(q24))/no alterations); higher hTERT expression; positive for CD90, CD73, HLA-ABC, CD166, negative for HLA-DR, CD34, CD19, CD14; comparable immunomodulatory capabilities on T-cell proliferation	EV concentration using 100 kDa Amicon^®^ units (Millipore, Darmstadt, Germany), purification performed by SEC, produced in hollow fiber bioreactors; cryo-TEM revealed the presence of a high number of vesicles ranging from 80 to 500 nm; CD63, CD81, and CD9 highly expressed, CD29 and CD44 showed high expression in both 3D- and 2D-produced EVs, CD29 expression was higher for 2D-derived EVs, while no significant differences were observed between 3D- and 2D-produced EVs in CD44 and other markers (e.g., CD146, CD42a, CD41b, CD105); able to induce HUVEC differentiation to tube-like structures in vitro in a dose-dependent manner	No expression of hTERT mRNA by qPCR detected in primary fibroblasts after incubation with iMSC-EVs
AT-MSCs from a patient with venous stasis ulcer (82 years old) and a healthy donor (22 years old) [70]	hTERT, pSV3-neo plasmids (ViaFect™ Transfection Reagent), single-cell clones, HATMSC1 (from ulcer patient), HATMSC2 (from healthy donor); positive for CD73, CD90, CD105, CD146, CD45, HLA-ABC antigens, negative for HLA-DR and CD45, minimal expression of CD34; repeated testing over the course of 12 months’ culturing showed no changes in expression profile; hypoxic conditions were used; did not engraft and form tumors in immunodeficient mice (also shown by histopathological analysis of skin and different organs), did not acquire anchorage-independent growth	Supernatant concentrated with Amicon^®^ Ultra 15 mL centrifugal 3 kDa filters; pro-angiogenic cytokines GRO, IL-6, IL-8, RANTES, and VEGF expressed, high levels of angiogenesis regulatory molecules IGF-1, MCP-1, MMP-1, TIMP-1, and TIMP-2; increased survival, viability, and proliferative potential in all skin-derived cells (MSU-1.1, HaCaT, HSkMEC.2), increased migratory potential of fibroblasts; dose-dependent angiogenic properties in vitro	Was not tested
AT-MSCs from a patient with venous stasis ulcer (82 years old) [71]		Microvesicles isolated through a sequence of centrifugations; average size 584.6 nm; internalization into the target cells; pro- and anti-angiogenic factors (e.g., IL-8, VEGF, TIMP-1, and TIMP-2), high concentration of EGF, FGF-2, and MCP-1, increased expression of pro-angiogenic miRNAs (miR-210, miR-296, miR-126, and miR-378), low expression of anti-angiogenic miRNA expression (miR-221, miR-222, and miR-92a); increased the proliferation of human HSkMEC.2 and fibroblasts, no effect on the proliferation of keratinocytes, improved the angiogenic properties of endothelial cells, depending on the dose	
AT-MSCs [72]	hTERT-immortalized AT-MSCs (SCRC-4000™ cell line, purchased from ATCC^®^), adapted to suspension culture (S-hMSCs); S-hMSCs had comparable growth kinetics to primary and immortalized AT-MSCs; doubling time of app. 55 h; retained close to 90% of CD73 and CD105 expression levels, the CD90 receptor being downregulated during the adherent to suspension adaptation process, upregulation of the transcripts coding for CD44, CD46, and CD47 compared to the expression levels in primary and immortalized AT-MSCs (by RNA sequencing)	EVs isolated via ultracentrifugation; mean size 153 nm; expressing CD63, CD81, TSG101; functional studies are planned	Not yet performed
AT-MSCs [73]	iMSCs purchased from ATCC^®^ (SCRC-4000^TM^); showed positive expression of CD29, CD44, CD73, CD90, CD105, absence of CD31, CD34, CD45, and HLA-DR; differentiation into adipocytes, osteocytes, and chondrocytes confirmed	Isolated using the Total Exosome Isolation Reagent (4478359, Invitrogen, Waltham, MA, USA) from iMSCs primed with serum derived from a non-human primate rheumatoid arthritis (RA) model; serum IL-4 and the proportion of Th2 (CD4+CD25+GATA3+) and M2 (CD11c-CD206+ of CD45+CD64+) cells were significantly increased compared to the control when sEVs-RA were administered to the collagen-induced arthritis model; sEVs-RA alleviated cartilage damage by significantly lowering the concentrations of pro-inflammatory cytokines (TNF-α, keratinocyte chemoattractant, IL-12p70)	Was not performed
AT-MSCs [74,75]	Expression of hTERT mRNA; nuclear hTERT immunoreaction; more than PD100, normal karyotype (with a clone with chromosomal aberrations after PD83), no signs of senescence by SA-bGal staining; retained expression of CD44, CD73, CD90, CD105, did not express CD3, CD14, CD19, CD34, CD45, HLA-DR; osteogenic and adipogenic differentiation; in vivo tumorigenicity was absent	Exo-spin Kit (EX01, Cell Guidance Sytems Ltd., Cambridge, UK) or ultracentrifugation followed by the PEG precipitation method applied for the enrichment of sEVs; remarkable number of sEVs with proper size (40–150 nm) and protein concentration produced, proper integrity, size distribution, and polydispersity index; positive for common exosomal markers, including CD63 and TSG101	Was not performed
AT-MSCs [76,77]	Overexpression of human TERT using the genetic construct pVLT-EF1a-hTERT-puroR with lentiviral packaging plasmids; iMSCs demonstrated senescence signs (increased PDT and beta-galactosidase activity) after 30 passages; retained immunophenotype, proliferative activity, differentiation potential, hormone sensitivity (responded to glutamate, GABA, dopamine, noradrenaline, angiotensin II, histamine, 5-HT, parathormone by an increase in cytoplasmic Ca^2+^ influx), and sensitization capacity until at least 17–26 passages	EVs concentrated 10-fold using ultrafiltration cartridges with a molecular weight cutoff (MWCO) of 1000 kDa (Millipore, Darmstadt, Germany); EVs from pMSC and iMSC did not differ significantly in concentration and size (NTA); the secretomes of pMSCs and iMSCs were 94.5% identical	iMSC secretome did not contain detectable levels of telomerase protein or nucleic acids encoding it, did not change the expression of pro- and anti-oncogenes in primary fibroblasts, did not induce fibroblast colony formation in a soft agar colony formation assay
Corneal stromal stem cells (CSSCs) from corneal limbs [78,79]	Primary CSSCs transfected with SV40 LT, pBSSVD2005 plasmids; maintained their morphology for over 15 passages; no karyotype study; maintained MSC surface antigen expression profile and multilineage differentiation capacity	Tangential flow filtration, concentration using an Amicon^®^ Ultra-15 100 kDA filter, purification and collection of 200 μL fractions using size exclusion and binding chromatography; size distribution evaluated by NTA indicated a mode size of 88.8 nm, cup-shaped morphology; the presence of sEV-specific tetraspanins CD63, CD81, and CD9, the presence of specific endosomal proteins Alix, TSG101, and Syntenin-1, the absence of the cell marker Calnexin; modulated the effects of Dex on gene expression in human primary TM cells with a marked impact on the expression of the ANGPTL7 gene	Not yet performed
Human hair follicle-derived mesenchymal-like stromal cells (HF-MSCs) [80]	Lentiviral particles with SV40 LT; 2 selected clones had faster proliferation at higher passages; CD73 expression remained very high, CD90 and CD105 levels were slightly reduced; retained MSC-like identity, morphology, multipotency, and the ability to differentiate into osteogenic, adipogenic, and chondrogenic lineages, although adipogenesis remained less efficient	Supernatants were centrifuged at 460 g for 10 min to eliminate cell debris; secretome analysis demonstrated that both clones maintained functional properties of primary HF-MSCs; “licensed” (after exposing MSCs to pro-inflammatory cytokines IFN-γ and TNF-α) conditioned media were used; key immunomodulatory mediators were present; inhibited peripheral blood mononuclear cell proliferation, promoted Treg differentiation, demonstrated strong antioxidant activity that protected skin cells from oxidative and hyperglycemic stress	Was not tested

## Data Availability

No new data were created or analyzed in this study. Data sharing is not applicable to this article.

## Abbreviations

The following abbreviations are used in this manuscript:

MSCs	mesenchymal stromal (stem) cells
ISCT	International Society for Cell and Gene Therapy
NK	natural killer
MHC	major histocompatibility complex
HLA	human leukocyte antigen
ECM	extracellular matrix
EVs	extracellular vesicles
MISEV	Minimal Information for Studies of Extracellular Vesicles
sEVs	small extracellular vesicles
MSC-(s)EVs	MSC-derived (small) EVs
CNS	central nervous system
LPS	lipopolysaccharides
ICPs	immune checkpoint proteins
ICPLs	immune checkpoint ligands
TE	tropoelastin
VEGF	vascular endothelial growth factor
AT-MSCs	adipose (tissue)-derived stem/stromal cells
SGD	serum- and glucose-deprived
hUCMSCs	human umbilical cord mesenchymal stem cells
HUVEC	human umbilical vein endothelial cells
BMSCs	bone marrow mesenchymal stem cells
XFM	xeno-free media
LIPUS	low-intensity pulsed ultrasound
CQAs	critical quality attributes
SOCRATES, ISEV, ISCT, and ISBT	four leading international scientific societies collaborating to standardize the characterization and therapeutic use of MSC-EVs
SOCRATES	Society for Clinical Research and Translation of Extracellular Vesicles Singapore
ISEV	International Society for Extracellular Vesicles
ISBT	International Society of Blood Transfusion
GMP	good manufacturing practice
HFB	hollow-fiber bioreactor
CMSCs	placental chorionic mesenchymal stromal cells
ESC	embryonic stem cell
iMSCs	immortalized MSCs
hTERT	human telomerase reverse transcriptase
NTA	nanoparticle tracking analysis
EM	electron microscopy
TEM	transmission electron microscopy
S-hMSCs	suspension culture human mesenchymal stromal stem cells
HPLC	high-performance liquid chromatography
ATCC^®^	American Type Culture Collection
CSSCs	corneal stromal stem cells
HF-MSCs	human hair follicle-derived mesenchymal-like stromal cells
imCMSCs	immortalized CMSCs
E1-MYC	ESC-derived monoclonal MSC line
pMSCs	primary MSCs
PD	population doubling
PDT	population doubling time
HATMSC	immortalized AT-MSCs
GRO	growth-regulated oncogene
IL	interleukin
IGF-1	insulin growth factor 1
MMP	matrix metalloproteinase
HEPC-CB.1	human endothelial progenitor cell line originating from cord blood
bFGF	basic fibroblast growth factor
sEVs-RA	MSC-derived sEVs primed with RA serum
FBS	fetal bovine serum
TGF-β1	transforming growth factor beta 1
HGF	hepatocyte growth factor
SASP	senescence-associated secretory phenotype
BDNF	brain-derived neurotrophic factor
SV40 LT	Simian virus 40 large T antigen
RT-PCR	reverse transcription polymerase chain reaction
DRG	dorsal root ganglion
TRAP	telomeric repeat amplification protocol
imCSSCs	immortalized corneal stromal stem cells
HF-MSCs	hair follicle-derived MSCs
DPCs	dermal papilla-derived stem cells
SPECT	single-photon emission computed tomography
PET	positron emission tomography
MRI	magnetic resonance imaging
CT	computed tomography
ELISA	enzyme-linked immunosorbent assays
SEC	size-exclusion chromatography
hT-HFF cells	hTERT-immortalized fibroblasts
AML	acute myelogenous leukemia
ROCK1	Rho kinase 1
